# Mesenchymal stem cell-derived extracellular vesicles subvert Th17 cells by destabilizing RORγt through posttranslational modification

**DOI:** 10.1038/s12276-023-00949-7

**Published:** 2023-03-24

**Authors:** Sunyoung Jung, Sunho Lee, Hyun Je Kim, Sueon Kim, Ji Hwan Moon, Hyunwoo Chung, Seong-Jun Kang, Chung-Gyu Park

**Affiliations:** 1 Department of Microbiology and Immunology, Seoul National University College of Medicine, Seoul, 03080 Korea; 2 Department of Biomedical Sciences, Seoul National University College of Medicine, Seoul, 03080 Korea; 3 Institute of Endemic Diseases, Seoul National University College of Medicine, Seoul, 03080 Korea; 4 Seoul National University Hospital, Seoul, Korea; 5 Transplantation Research Institute, Seoul National University College of Medicine, Seoul, 110-799 Korea; 6 Samsung Genome Institute, Samsung Medical Center, Seoul, Korea; 7 Cancer Research Institute, Seoul National University College of Medicine, Seoul, 03080 Korea; 8 BK21Plus Biomedical Science Project, Seoul National University College of Medicine, Seoul, 03080 Korea; 9 Biomedical Research Institute, Seoul National University College of Medicine, Seoul, 03080 Korea

**Keywords:** T cells, Mesenchymal stem cells

## Abstract

Mesenchymal stem cell (MSC)-derived small extracellular vesicles (MSC-sEVs) are known to exert immunosuppressive functions. This study showed that MSC-sEVs specifically convert T helper 17 (Th17) cells into IL-17 low-producer (ex-Th17) cells by degrading RAR-related orphan receptor γt (RORγt) at the protein level. In experimental autoimmune encephalomyelitis (EAE)-induced mice, treatment with MSC-sEVs was found to not only ameliorate clinical symptoms but also to reduce the number of Th17 cells in draining lymph nodes and the central nervous system. MSC-sEVs were found to destabilize RORγt by K63 deubiquitination and deacetylation, which was attributed to the EP300-interacting inhibitor of differentiation 3 (Eid3) contained in the MSC-sEVs. Small extracellular vesicles isolated from the Eid3 knockdown MSCs by Eid3-shRNA failed to downregulate RORγt. Moreover, forced expression of Eid3 by gene transfection was found to significantly decrease the protein level of RORγt in Th17 cells. Altogether, this study reveals the novel immunosuppressive mechanisms of MSC-sEVs, which suggests the feasibility of MSC-sEVs as an attractive therapeutic tool for curing Th17-mediated inflammatory diseases.

## Autoimmune disease: Stem cell vesicles have therapeutic potential

Tiny membrane-bound vesicles derived from certain stem cells could provide effective therapy for a range of autoimmune conditions. Adult or mesenchymal stem cells (MSCs) in the bone marrow, fat, and other tissues have shown promise in immunotherapy which is associated with the secretion of biomolecule-laden structures known as extracellular vesicles. Chung-Gyu Park, Seoul National University, South Korea, and colleagues have now helped clarify a likely therapeutic mechanism. The authors showed marked relief of symptoms in a mouse model of autoimmune disease upon treatment with MSC-derived vesicles and linked this effect to reduced levels of immune cells known as TH17 cells. Further analysis revealed that these vesicles selectively suppress the production and activation of TH17 cells, which generally play a prominent role in exacerbating various autoimmune diseases including lupus and psoriasis.

## Introduction

Mesenchymal stem cells (MSCs) have been clinically used for preventing and treating various autoimmune diseases or graft-versus-host disease (GVHD) due to their potent immunomodulatory properties^1^. MSCs not only inhibit T-cell responses but also modulate the functions of B cells, natural killer cells, and dendritic cells^2^. Additionally, MSCs have been reported to regulate the balance between Th17 and Treg cells^3^. In particular, MSCs exert their immunosuppressive effect via cell-to-cell interactions and soluble factors, such as iNOS, IL-10, TGF-β, HGF, PGE2, and IDO^4^.

However, infusing MSCs has certain drawbacks, including a short survival time and the possibility of becoming trapped in the lung^5^. Due to their small size, small extracellular vesicles (sEVs) from mesenchymal stem cells can bypass most physiological barriers and reach their target tissues^6^. Consequently, sEVs can be utilized in clinical settings by filtration-based sterilization. Thus, MSC-derived small extracellular vesicles (MSCs-sEVs) provide significant clinical advantages.

Small extracellular vesicles are nanosized lipid bilayer membrane vesicles that are secreted from cells and serve as mediators of cell-to-cell communication^7^. MSCs can release extracellular vesicles, exerting immunomodulatory and regenerative effects^8^. Extracellular vesicles contain various molecules, including signal peptides, mRNAs, microRNAs, and lipids^9^. Recent studies suggest that extracellular vesicles produced from MSCs are crucial for mediating the biological function of MSCs^10^. Since MSCs actively secrete extracellular vesicles, several studies have reported the efficacy of MSC-sEVs in various disease models^11^. The mechanisms by which MSCs regulate the activation and differentiation of T cells were investigated, and it was discovered that the soluble mediators from MSCs suppress T-cell proliferation by inducing the IL-10- and matrix metalloprotease-mediated cleavage of IL-2Rα^12,13^. In these studies, MSC-sEVs were found to suppress T-cell proliferation by inducing cell cycle arrest through p27kip1/Cdk2 signaling^14^.

Depending on the expression of specific cytokines and functions, CD4^+^ T cells are subdivided into Th1 (T helper type 1), Th2 (T helper type 2), Th17 (T helper 17), and CD4^+^ CD25^+^ immunoregulatory T cells^15^. Th17 cells that produce IL-17 not only defend the host against pathogens but also trigger inflammatory and autoimmune diseases such as psoriasis, multiple sclerosis, chronic inflammatory bowel disease, and systemic lupus erythematosus^16^. The inhibition and regulation of Th17 cells are believed to be effective strategies for treating Th17-mediated inflammatory and autoimmune diseases^17^. Therefore, several targeted therapeutic strategies utilizing the inhibition and neutralization of Th17 cell-related cytokines and specific transcription factors such as RORγt are being investigated^18^.

MSC-sEVs alleviate autoimmune diseases by regulating the balance between Th17 and Treg cells^19^. However, the mechanisms by which MSC-sEVs regulate T-cell activation and Th17 differentiation remain unclear.

This study showed that MSC-sEVs can inhibit Th17 differentiation and convert differentiated Th17 cells into IL-17 low-producing Th17 cells (ex-Th17) by proteasomal degradation of RORγt via reduction of K63-linked polyubiquitination and acetylation. Eid3 in MSC-EVs is required and sufficient for K63-linked deubiquitination and deacetylation by suppressing p300, a ubiquitin ligase known to have acetyltransferase activity^20,21^. MSC-sEVs specifically suppressed the Th17 lineage in the murine EAE model. This study revealed the mechanism underlying the regulation of Th17 cell differentiation by MSC-sEVs, which could contribute to the development of therapeutic modalities targeting Th17-mediated inflammatory and autoimmune diseases using MSC-EVs.

## Materials and methods

### Mice

For this study, female C57BL/6NHsd mice aged 8 weeks were purchased from KOATECH (Seoul, Korea) and used within 1 week. The animals were housed in an animal facility at the Seoul National University College of Medicine, Seoul, Republic of Korea. All experiments were performed in accordance with the approved IACUC protocol (approval number SNU-170221-3-1) and complied with the institutional guidelines.

### Cell culture

Murine bone marrow-derived mesenchymal stem cells (MSCs) were freshly isolated from long bones such as femurs and tibias. Isolated MSCs were characterized to confirm that they did not express hematopoietic stem cell markers and had a compatible phenotype to that of BM-originated MSCs^22^. MSCs were cultured in Dulbecco’s modified Eagle’s medium (DMEM) supplemented with 10% fetal bovine serum (FBS) and 50 µg/mL gentamycin in a humidified 5% CO_2_ atmosphere at 37 °C.

### Isolation and purification of extracellular vesicles

The culture medium was replaced with FBS-sEVs-depleted culture medium (EDCM) when the MSCs reached 80% confluency. The culture supernatant of MSCs was harvested after 48 h. The extracellular vesicles were purified by differential ultracentrifugation as follows: (1) The collected supernatant was centrifuged at 500 × *g* and 4 °C for 10 min. (2) The pellet was discarded, and the supernatant was centrifuged at 2000 × *g* and 4 °C for 10 min. (3) The pellet was discarded, and the supernatant was filtered through a 0.22-μm filter (Merck, NJ, USA) and ultracentrifuged at 100,000 × *g* and 4 °C for 75 min (Beckman Coulter, CA, USA) using a 45 Ti fixed rotor (Beckman Coulter). The pellet at the bottom of the centrifuge tube was washed once by resuspending it in 60 ml of EDCM. For further purification, the pellet was resuspended in 25 ml of PBS. The diluted pellet was gently layered on 4 ml of Tris/sucrose/D_2_O solution in an SW28 tube (Beckman Coulter) and centrifuged at 100,000 × *g* at 4 °C for 75 min. The bottom sucrose fraction was collected, diluted with 60 ml of PBS, and centrifuged at 100,000 × *g* at 4 °C for 75 min. The purified extracellular vesicle pellet was resuspended in 100–150 μl of PBS and frozen at −80 °C for further experiments.

### Sucrose density gradient ultracentrifugation

The sucrose solutions were prepared by diluting 10 M sucrose stock solution with HEPES buffer. A continuous sucrose gradient solution was prepared by mixing 2.5 ml of 2 M sucrose solution and 2.5 ml of 0.25 M sucrose solution in a gradient maker (Sigma, MO, USA). The MSC-sEVs were resuspended in 2.5 M sucrose solution and layered on the bottom of the SW28 tube. The sucrose gradient solution was gently layered on the MSC-EV suspension and centrifuged at 100,000 × *g* for 3 h. The centrifuged solution was fractionated by 1 ml and aliquoted into microcentrifuge tubes. The specific gravity of each fraction was measured using a densimeter (Kyoto Electronics, Mexico City, Mexico). Each fraction was analyzed using SDS‒PAGE, followed by western blotting using mouse anti-Alix (Cell Signaling Technology, MA, USA) and mouse anti-Calnexin (BD Biosciences, NJ, USA) antibodies.

### Quantification of extracellular vesicles

Total protein in the extracellular vesicles was measured by the bicinchoninic acid (BCA) assay according to the manufacturer’s protocol (Pierce, IL, USA). The number of extracellular vesicles was determined using the EXOELISA kit (System Biosciences, CA, USA) according to the manufacturer’s protocol. The absorbance was measured at 450 nm. The concentration of extracellular vesicles was determined using a linear equation calculated from the standard curve using calibrated standards provided in the EXOELISA kit (System Biosciences, CA, USA).

### Western blot analysis

The extracellular vesicles were heated in SDS sample buffer at 100 °C for 10 min, separated using 12% SDS‒PAGE, and transferred to PVDF membranes (Merck, NJ, USA) using an electrotransfer system (300 mA, 4 °C, 1 h). The PVDF membrane was then incubated at RT for 1 h in PBS containing 0.5% Tween-20 and 5% nonfat dry milk for blocking. After a brief wash with 0.5% Tween-20 PBS (PBST), the membrane was probed with anti-calnexin (Santa Cruz, CA, USA), anti-Alix (Santa Cruz), anti-phospho-STAT3, anti-STAT3 (Cell Signaling Technology), anti-p300 (Santa Cruz), anti-K63 ubiquitin (Abcam, Cambridge, UK), anti-Eid3 (Abcam), anti-RORγt (Santa Cruz), anti-acetyl-K-HRP (Immunechem, Burnaby, Canada), and anti-RORγt (Invitrogen, CA, USA) antibodies in PBST overnight at 4 °C. The membrane was washed three times with PBST and incubated with HRP-conjugated IgG (Jackson Immunoresearch Laboratories, PA, USA) antibody at RT for 30 min. After five washes with PBST, the signal was developed with enhanced chemiluminescence reagents (Pierce Biotechnology) and acquired using LAS4000 (GE Healthcare, IL, USA).

### Transmission electron microscopy

The purified extracellular vesicles were fixed with 2.5% glutaraldehyde for 4 h and postfixed with 1% osmium tetroxide at 4 °C for 1 h. The fixed extracellular vesicles were embedded in epoxy resin after dehydration by incubation in a graded series of ethanol solutions (30%, 50%, 70%, 80%, 90%, and 100%) for 15 min. Subsequently, ultrathin sections were prepared using an ultramicrotome equipped with a diamond knife (Diatome Ltd., Nidau, Switzerland). Heavy metal staining was performed with 4% uranyl acetate and lead citrate (Sigma). The observations were made using an H-600 electron microscope (Hitachi High-Technologies Europe GmbH, Krefeld, Germany).

### Treg, Th1, and Th17 polarization

Naive CD4^+^ T cells (CD4^+^CD62L^+^CD44^-^CD25^-^) were isolated using MACS negative selection (Miltenyi Biotec, Bergisch Gladbach, Germany). The mouse splenocytes were labeled with a cocktail of biotinylated monoclonal antibodies against non-CD4^+^ T cells (CD8a, CD11b, CD11c, CD19, CD45R (B220), CD49b (DX5), CD105, anti-MHC class II, Ter-119, and TCRγ/δ) as primary labeling reagents, and anti-biotin microbeads were added as secondary labeling reagents. The labeled splenocytes were added to a MACS® column subjected to the magnetic field of a MACS separator. The unlabeled cells that passed through the column were obtained. The cells were labeled with biotin-conjugated anti-CD25 (Miltenyi Biotec) and anti-CD44 (Miltenyi Biotec) antibodies. The labeled cells were depleted by retention in the column, and the cells that passed through were used as the naive CD4^+^ T cells.

Isolated naive CD4^+^ T cells (5 × 10^4^) were added to each well of a 96-well plate coated with anti-CD3e (5 μg/ml) and anti-CD28 (5 μg/ml) antibodies (eBioscience, CA, USA). The differentiation of the Treg, Th1, and Th17 cells was induced as follows: For Treg differentiation, rmIL-2 (100 U/ml) and TGF-β (5 ng/ml) were added and incubated for 3 days at 37 °C in a humidified 5% CO_2_ incubator. For Th1 differentiation, mIL-12 (20 ng/ml), neutralizing anti-IL-4 antibody (10 μg/ml), and rmIL-2 (50 units/ml) were added and incubated for 5 days at 37 °C in a 5% CO_2_ humidified incubator. On Day 3, the cells were restimulated with mIL-12 (20 ng/ml), neutralizing anti-IL-4 (10 μg/ml), and rmIL-2 (50 units/ml). For Th17 differentiation, mIL-6 (20 ng/ml), TGF-β (1 ng/ml), and neutralizing anti-IL-4/IFN-γ antibody (10 μg/ml) were added and incubated for 5 days.

### CFSE staining

Approximately 1 × 10^7^ cells were suspended in 10 ml of sterilized PBS. Carboxyfluorescein succinimidyl ester (CFSE, eBioscience) was added and mixed gently to achieve a final concentration of 1.5 μM. The cells were incubated in a water bath (37 °C) for 5 min and on ice for 5 min. Then, the CFSE-tagged cells were washed twice with PBS and once with DMEM.

### Cytometric bead array (CBA) assay

The amounts of TGF-β, IL-17, IL-2, IL-4, IL-6, and IFN-γ in the culture supernatant were measured using the mouse Th1/Th2/Th17 CBA detection system (BD Biosciences) following the manufacturer’s instructions. Standard curves were generated for each cytokine, and the concentration of each cytokine in the culture supernatant was measured by extrapolation from an appropriate standard curve. All samples were analyzed by flow cytometry (BD Biosciences) and BD CBA software (FCAP Array™ v1.0.2).

### Confocal microscopy

Extracellular vesicles (5 μg) were suspended in binding buffer (System Biosciences), added to confocal dishes, and incubated overnight at 37 °C. The supernatant was discarded, and the dish was washed for 5 min with 100 μl of 1× wash buffer three times (System Biosciences). The rat anti-mouse CD9 monoclonal antibody (eBioscience) and Alexa-fluor 555 conjugated goat anti-rat antibody (eBioscience) were added to the dish and incubated at RT for 1 h. The dish was washed three times for 5 min with 100 μl of 1× wash buffer. The images were acquired using an FV1000 confocal microscope (Olympus, Tokyo, Japan) and were analyzed using OLYMPUS FLUOVIEW software.

### Induction of experimental autoimmune encephalomyelitis (EAE) and administration of MSC-sEVs

Myelin oligodendrocyte glycoprotein (MOG)-induced EAE using MOG35–55 peptides was performed as described previously^23^. The severity of the disease was scored by two independent investigators as follows: 0, no clinical signs; 0.5, partial loss of tail tonicity; 1, complete loss of tail tonicity; 2, flaccid tail and abnormal gait; 3, hind leg paralysis; 4, hind leg paralysis with hind body paralysis; 5, hind and foreleg paralysis; and 6, death. Following the appearance of the EAE symptoms, the scored mice were stratified and assigned to separate test groups to generate equally weighted average disease scores before the experimental interventions*.*

### Isolation of immune cells from the spinal cord and brain

Mice were sacrificed at the given time, and the brain and spinal cord were isolated according to a previously reported protocol^24^. The brain and spinal cord were dissected, resuspended in 3 ml of RPMI, and homogenized for 1 min using a Dounce homogenizer. RPMI was added to a final volume of 7 ml of the cell suspension. A Percoll (GE Healthcare) density gradient protocol was conducted^25^ to isolate the infiltrated immune cells. Briefly, 70% and 30% Percoll solutions were prepared by dilution with 1× HBSS. The homogenized brain and spinal cord were resuspended in 3 ml of 30% Percoll solution. Ten milliliters of homogenized cell suspension was layered slowly on top of 70% Percoll in a 2-ml tube and centrifuged at 500 × *g* for 30 min. Three milliliters of 70–30% interphase was collected and washed with 8 ml of 1× HBSS three times by centrifugation at 500 × *g* for 7 min. The purified immune cells were resuspended in 1 ml of complete medium for further experiments (approximately 3–5 × 10^5^ immune cells were isolated per mouse).

### ELISpot

To coat the capture antibodies, 100 μl of each antibody, including anti-IFN-γ (6 μg/ml), anti-IL-6 (6 μg/m), and anti-IL-17 (6 μg/ml) (all from eBioscience), was aliquoted into each well of MultiScreen-hemagglutinin plates (Sigma) and incubated overnight at 4 °C. After briefly washing the plates with PBS, 5 × 10^5^ splenocytes were added to each well and incubated for 48 h at 37 °C. The plates were washed three times with PBST. Biotin-conjugated detection antibodies (100 μl), including anti-IFN-γ (6 μg/ml), anti-IL-6 (6 μg/ml), and anti-IL-17 (6 μg/ml), were added and incubated at 4 °C for 24 h. The plates were washed three times with PBST. Streptavidin-horseradish peroxidase (100 μl, 1:1000) was added and incubated at RT for 2 h. The plates were washed five times with PBST, and the color reaction was developed using 3-amino-9-ethyl carbazole (Sigma) as the substrate. The number of spots was determined using an ELISA reader (AID, Strassberg, Germany).

### Lentiviral vector transduction

#### Knockdown of Eid3 expression in MSCs

EID3 shRNA lentiviral particles (ORIGENE, MD, USA) were transduced, and the MSCs (2 × 10^5^ cells/well) were cultured in 12-well culture plates at 37 °C for 24 h. The EID3 shRNA lentiviral particles were preincubated with polybrene (5 μg/ml) at 37 °C for 2 h. Five multiplicities of infection (MOIs) of EID3 shRNA lentiviral particles were added to the 12-well plates and incubated at 37 °C for 12 h. The medium was replaced with fresh medium and incubated for 24 h. After incubation, the medium was replaced with fresh medium containing 2 μg/ml puromycin, and MSCs resistant to puromycin were selected as EID3 shRNA lentivirus-transduced cells.

#### Forced expression of Eid3 in T cells

T cells were transduced with green fluorescent protein (GFP)-tagged Eid3-ORF lentiviral particles (ORIGENE). Briefly, the Eid3-ORF lentiviral particles were incubated with polybrene (5 μg/ml) at 37 °C for 2 h. The lentiviral particles were added to the Th17 cells. After incubation at 37 °C for 12 h, the medium was replaced with fresh medium and incubated for 24 h. Transduced Th17 cells were selected for GFP expression by flow cytometry.

### Quantitative reverse transcriptase polymerase chain reaction (qRT‒PCR)

RNA was isolated from the extracellular vesicles using the Total Extracellular Vesicle RNA & Protein Isolation Kit (Invitrogen) according to the manufacturer’s protocol. Total RNA was isolated from Th17 cells using TRIzol reagent (Invitrogen) according to the manufacturer’s protocol. cDNA was synthesized from the isolated mRNA using the SuperScript™ III First-Strand Synthesis System Kit (Invitrogen). The forward and reverse primers for qPCR were designed using the OligoPerfect Primer Designer (Thermo Fisher, MA, USA). The sequences of the primers were as follows: forward (5ʹ-GAGAGAAGTGCCGCAAAATC-3ʹ) and reverse (5ʹ-TCGCCTTCTCTTTACCCAGA-3ʹ) for Eid3; forward (5ʹTGCAAGACTCATCGACAAGG 3ʹ) and reverse (5ʹ AGGGGATTCAACATCAGTGC 3′) for RORγt; forward (5′ CAGACTACCTCAACCGTTCCAC 3′) and reverse (5′TCCAGCTTTCCCTCCGCATTGA 3′) for IL-17A. The expression level was assessed using SYBR™ Select Master Mix (Invitrogen). GAPDH was used as an internal control.

### Immunoprecipitation

Anti-RORγt antibody (Santa Cruz) was used for immunoprecipitation. The cell lysates (500 μg of cell lysate in 500 μl of RIPA, without SDS) were incubated with 30 μl of protein G bead slurry (GE Healthcare) and 10 μg of normal IgG (Invitrogen) for 1 h at 4 °C for preclearing. The supernatant was treated with 2 μg of anti-RORγt antibody overnight at 4 °C with gentle agitation. Then, 30 μl of bead slurry was added to the lysates, and the sample was incubated for 4 h at 4 °C with gentle agitation. After microcentrifugation at 3000 × *g* at 4 °C for 10 min, the supernatant was discarded, and the pellet was washed five times using RIPA buffer (without SDS). Twenty microliters of the 2× sample buffer was added to the bead pellet and boiled for 5 min at 100 °C. The supernatant was used for western blot analysis.

### Microarray analysis of mRNA

Naive CD4^+^ T cells were isolated using a magnetic separator (Miltenyi Biotec). Th17 polarization was induced in the presence or absence of MSC-sEVs. Total RNA was extracted from polarized T cells (Invitrogen) using the same methods described above. The Affymetrix microarray (Mouse Gene 1.0 ST Array) was conducted by Biocore (Seoul, Korea). The microarray data were analyzed to identify the differentially expressed genes (DEGs) between Th17 and MSC-sEVs-treated Th17 cells. GeneSpring GX 12.1 (Agilent, Santa Clara, CA) was used to identify the DEGs, and the cutoff values for the *P* value and log2 fold change were 0.05 and 0.5, respectively. A volcano plot for differential expression visualization was generated using the EnhancedVolcano R package with a log2 fold change cutoff of 0.5 and a *p* value cutoff of 0.05. The DEGs were annotated by searching the Gene Ontology database using AmiGO 2 software (Eddyfi Technologies, Québec, Canada).

### Statistical analysis

The results are expressed as the mean ± SEM. The statistical significance was determined by Student’s *t* test or two-way ANOVA, followed by Tukey’s post hoc test to analyze the clinical scores. Statistical significance was set at *P* < 0.05.

## Results

### Characterization of MSC-EVs

**Fig. 1:**
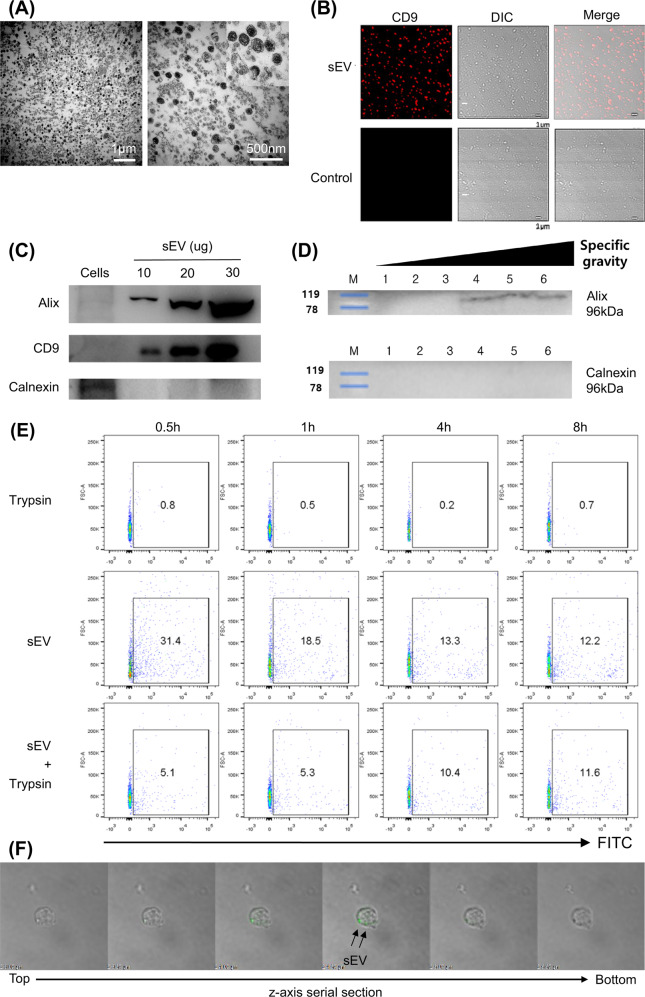
MSC-sEVs were characterized and were taken up by T cells. **A** Transmission electron microscopy (TEM) images of MSC-sEVs. The sEVs were observed with low power (20,000×, left) and high power (60,000×, right). sEVs exhibited a lipid bilayer structure and cup-shaped morphology. Scale bars, 1 µm (left) and 500 nm (right). **B** sEVs were visualized under a confocal microscope. sEVs were detected with confocal microscopy by Alexa Fluor 555 bound to the murine CD9 and DIC (differential interference contrast) channel (10,000×). **C** Immunoblots of MSC-sEVs lysates against Alix, CD9, and calnexin. The EV-specific markers Alix and CD9 were detected in proportion to the number of proteins. In contrast, calnexin, an ER membrane-bound protein, was not detected in sEVs. **D** Determination of the specific gravity. MSC-sEVs were resuspended in 2.5 M sucrose-containing HEPES buffer before ultracentrifugation. A 0.25–2 M sucrose gradient was applied to the suspension (fraction 1–6 = 1.05–1.19 g/ml; 1 = 1.05 g/ml, 2 = 1.10 g/ml, 3 = 1.13 g/ml, 4 = 1.15 g/ml, 5 = 1.1.7 g/ml, and 6 = 1.19 g/ml). The range of the specific density of sEVs measured by sucrose density gradient centrifugation corresponded to 1.15–1.19 g/ml. **E** Evaluation of EV internalization into T cells. The CD4+ T cells were cocultured with MSC-sEVs labeled with 5 µM CFSE and were trypsinized to remove surface-bound sEVs. The uptake of MSC-sEVs by T cells at different time points was analyzed by FACS. Representative results are shown here. **F** Confocal microscopy image of MSC-sEVs. CD4+ T cells were incubated with GFP-tagged MSC-sEVs for 17 h, and trypsin was added to remove surface-bound sEVs before observation. Z-axis serial sections of confocal microscopy revealed the ingestion of MSC-sEVs by T cells.

sEVs from the cultured supernatant of MSCs were purified by differential ultracentrifugation and characterized as described in the Materials and Methods. Transmission electron microscopy (TEM) revealed cup-shaped, lipid bilayer membranous particulates with an average diameter of 50–120 nm (Fig. 1A) that were positive for CD9 in confocal microscopy analysis, as shown in Fig. 1B. These features were comparable to those previously reported for sEVs^26^. The sEVs were then subjected to western blot analysis using ALIX and CD9 as extracellular vesicle-specific markers. As shown in Fig. 1C, ALIX and CD9 were detected in the pellet fraction in a dose-dependent manner, whereas calnexin, a marker for the endoplasmic reticulum, was not detected, confirming the characteristics of sEVs. The range of specific density of the sEVs measured by sucrose density gradient centrifugation corresponded to 1.15–1.19 g/ml (Fig. 1D). The quantification of sEVs was assessed by the BCA assay and ELISA (Supplementary Fig. 1).

### MSC-sEVs bound to naive CD4^+^ T cells and were internalized

To define the interaction between MSC-sEVs and naive T cells, MSC-sEVs were labeled with CFSE and cocultured with CD4^+^ T cells. T cells were analyzed by FACS with or without trypsinization following 0.5, 1, 4, and 8 h of incubation. As shown in Fig. 1E, after 0.5 h of incubation, 31.4% of T cells exhibited green fluorescence positivity. With trypsinization, 5.1% of T cells were positive, indicating that the internalization of MSC-sEVs into the cytosol of T cells occurred in 5.1% out of 31.4% of T cells. After 8 h of incubation, 12.2% of T cells were positive for green fluorescence, and trypsinization did not significantly alter the positivity, suggesting that at a given ratio of MSC-sEVs to CD4+ T cells, the MSC-sEVs may be internalized in approximately 12% of T cells. The interaction between MSC-sEVs and T cells was also observed under a confocal microscope. The MSC-sEVs were tagged with eGFP using the lipid-raft-GPI-eGFP plasmid (Supplementary Fig. 2), utilizing the fact that extracellular vesicles are secreted from the cells of origin via exocytosis at lipid rafts. Then, the naive CD4^+^ T cells were cocultured with eGFP-tagged MSC-sEVs. As depicted in Fig. 1F and Supplementary Video 1, consecutive Z-axis sections of the confocal microscopic analysis revealed green fluorescence within the T cells. Taken together, these data show that MSC-sEVs can bind to and be internalized by T cells.

### MSC-sEVs specifically suppressed the differentiation of naive CD4^*+*^ T cells into Th17 cells

**Fig. 2:**
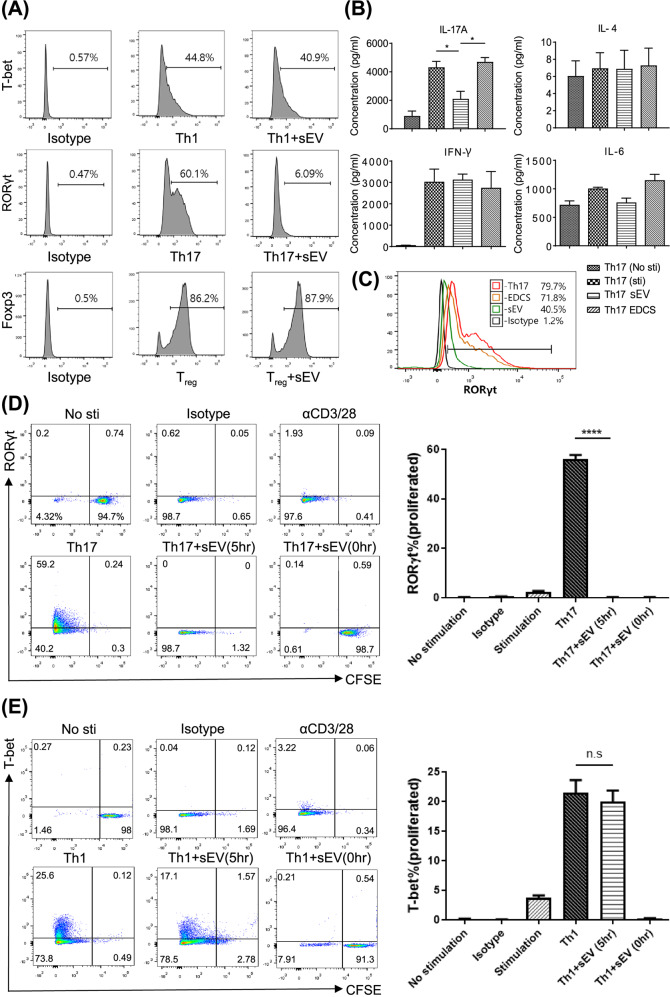
MSC-sEVs specifically inhibited Th17 cells by targeting RORγt. **A** Effect of MSC-sEVs on Th1, Th17, and Treg cell differentiation. Naïve CD4+ T cells differentiated into Th1, Th17, and Treg cells in the presence of MSC-sEVs. The expression of transcription factors (T-bet for Th1, RORγt for Th17, and Foxp3 for Treg) was analyzed by FACS. MSC-sEVs significantly reduced the number of RORγt+ T cells but not Foxp3+ and T-bet+ T cells. **B** Effect of MSC-sEVs on the cytokine secretion by Th17 cells. The levels of IL-17A, IL-4, IFN-γ, and IL-6 secreted from Th17 cells cocultured with MSC-sEVs were measured by the cytometric bead array (CBA) assay. Treatment with sEVs decreased the production of IL-17 but not other cytokines, including IFN-γ, IL-4, and IL-6, by Th17 cells. **C** Analysis of RORγt expression levels after Th17 differentiation in the presence of MSC-sEVs. sEVs-depleted culture serum (EDCS) and sEVs were used to treat differentiated Th17 cells. EDCS failed to reduce the number of RORγt+ T cells, indicating that MSC-sEVs were key players in the suppression of Th17 differentiation. **D**, **E** Assessment of the proliferation level of Th1 and Th17 cells treated with MSC-sEVs. Naive CD4+ T cells were labeled with CFSE (5 µM, 5 min) and differentiated into Th1 and Th17 cells under MSC-sEVs treatment conditions. sEVs specifically suppressed the differentiation of Th17 cells, and their mechanisms were independent of cell cycle arrest. A representative image from the three independent assays is shown in the dot plot. The percentages of RORγt- and T-bet-positive cells among the proliferating cells are shown in the plot. The mean of triplicates is displayed as a bar graph, and the error bars indicate SEM. ****p ≤ 0.0001.

In the presence or absence of MSC-sEVs, naive CD4^+^ T cells purified from C57BL/6 splenocytes were induced to differentiate into Th1, Th17, and Treg cells. As shown in Fig. 2A, MSC-sEVs significantly reduced the number of RORγt^+^ T cells but not Foxp3^+^ or T-bet^+^ T cells. The reduction in RORγt^+^ T cells resulted in a significant decrease in the production of IL-17 but not other cytokines, including IFN-γ, IL-4, and IL-6, as shown in Fig. 2B. The MSC culture supernatant deprived of MSC-sEVs (EDCS) failed to reduce the number of RORγt^+^ T cells (Fig. 2C), confirming that MSC-sEVs were the key player in suppressing Th17 differentiation. A previous study demonstrated that MSC-sEVs suppress T-cell proliferation via cell cycle arrest^14^. Therefore, to determine whether the suppression of Th17 differentiation is dependent on cell cycle arrest, a CFSE-labeled proliferation study was performed. Isolated naive CD4^+^ T cells were labeled with CFSE and placed under either Th17 or Th1 differentiation conditions. The MSC-sEVs were added from the onset of differentiation and 5 h after the initiation of differentiation. The proliferation of the T cells was assessed by CFSE dilution, and Th17 or Th1 differentiation was assessed based on the expression of RORγt or T-bet by flow cytometry. As shown in Fig. 2D, 98.7% of Th17-differentiated T cells did not proliferate or express RORγt when the MSC-sEVs were introduced at 0 h. However, when the MSC-sEVs were added 5 h after differentiation was initiated, 98.7% of the T cells were found to successfully proliferate but did not express RORγt, suggesting that the reduction in RORγt expression was independent of cell cycle arrest. Under Th1 differentiation conditions, approximately 91.3% of T cells failed to proliferate or express T-bet when MSC-sEVs were added at 0 h. However, when MSC-sEVs were added 5 h after the initiation of differentiation, 95.5% of T cells proliferated, and the expression of T-bet (17.1%) was not suppressed (Fig. 2E). These results suggested that the suppression of Th17 differentiation by MSC-sEVs was specific and mediated by mechanisms independent of cell cycle arrest. To determine whether these effects were common to sEVs regardless of their source, the same experiments were performed with fetal bovine serum-derived sEVs. As shown in Supplementary Fig. 3, neither suppression of T-cell proliferation nor inhibition of Th17 differentiation was observed.

### MSC-sEVs degraded RORγt at the protein level through K63-linked deubiquitination, resulting in the reduced production of IL-17 in CD4^+^ T cells polarized to Th17 cells

**Fig. 3:**
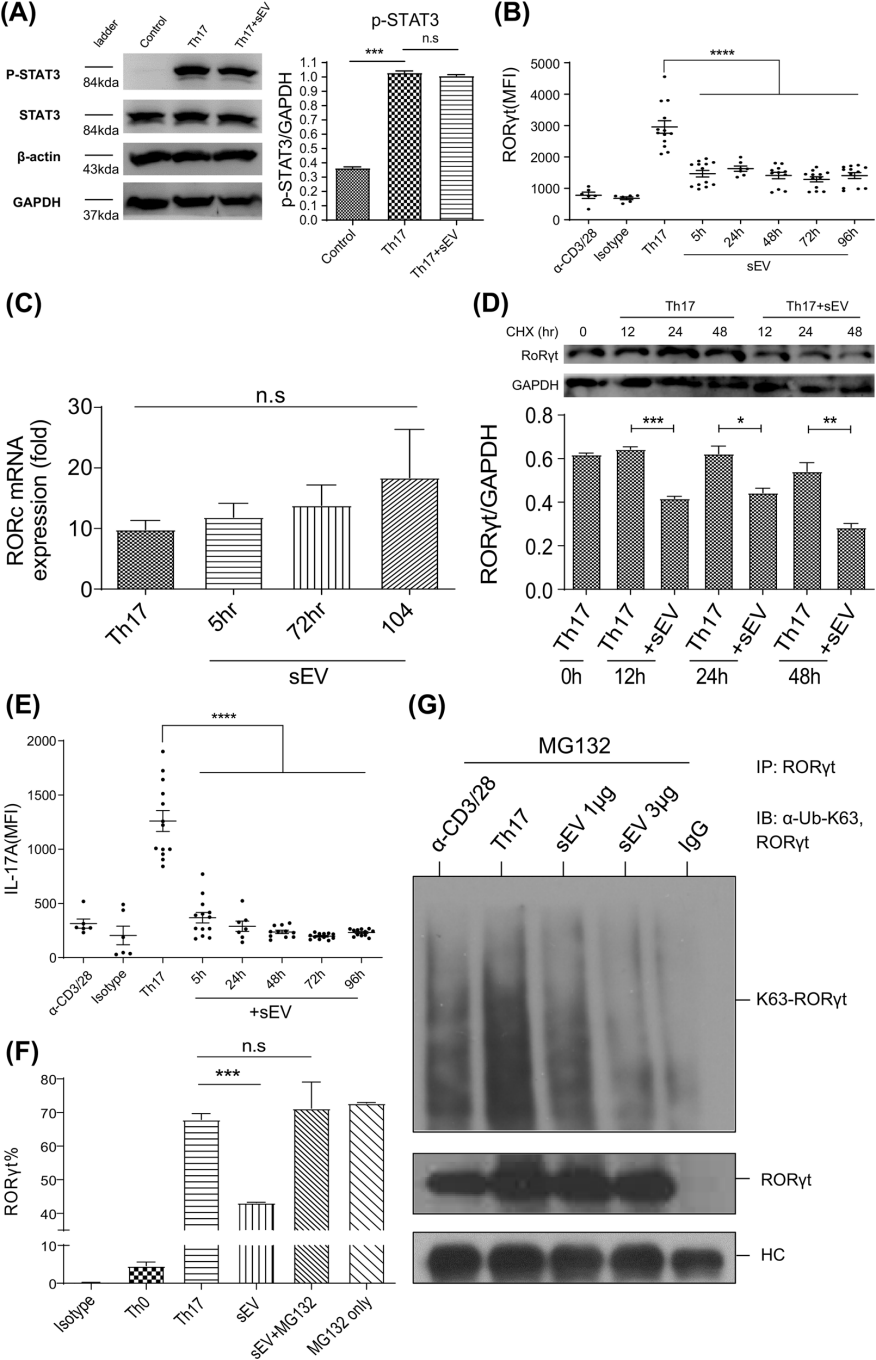
MSC-sEVs destabilized RORγt at the protein level in Th17 cells. **A** The phosphorylation level of STAT3 examined by immunoblotting. The normalized level of phospho-STAT3 (P-STAT3) was measured using individual band intensity (p-STAT3/STAT3 ratio, right panel). MSC-sEVs did not affect the phosphorylation of STAT3, indicating that MSC-sEVs regulate the expression of RORγt downstream of STAT3 phosphorylation. Representative images from the three independent immunoblots are shown. The mean of triplicates is shown as a bar graph, and the error bars indicate SEM. ***p ≤ 0.001. **B** Mean fluorescence intensity (MFI) of RORγt in MSC-sEVs-treated Th17 cells. MSC-sEVs were added at various time points after the initiation of Th17 differentiation (5, 24, 48, 72, and 96 h). The MFI of RORγt assessed by flow cytometry at 120 h of differentiation significantly decreased. **C** RORc mRNA expression levels were confirmed using real-time PCR. **D** Cycloheximide chase analysis of RORγt protein degradation. MSC-sEVs and 0.5 μg/ml cycloheximide were added to the T cells at 12, 24, and 48 h before the endpoint of Th17 differentiation (5 days). Zero hours indicates the positive control, which was not treated with MSC-sEVs and cycloheximide. Western blotting was conducted to visualize the expression level of RORγt. MSC-sEVs significantly reduced RORγt in the absence of de novo translated RORγt. A representative image from the three independent western blots. The RORγt/GAPDH ratio is shown as a bar graph. *p ≤ 0.05; **p ≤ 0.01; ***p ≤ 0.001. **E** Assessment of IL-17 expression levels using intracellular cytokine staining of MSC-sEVs-treated Th17 cells. MFI of IL-17A expressed in differentiated Th17 cells is shown with varying MSC-sEVs treatment time points. Reduction of RORγt by MSC-sEVs treatment resulted in a decrease in IL-17 production by Th17 cells. ****p ≤ 0.0001. **F** The ratio of RORγt (+) CD4+ T cells under Th17 polarizing conditions following treatment with MSC-sEVs. MG132 (2 μM). The proteasome inhibitor inhibited the reduction in the ratio of RORγt (+) cells mediated by MSC-sEVs. The mean of triplicates is shown as a bar graph, and the error bars indicate SEM. **G** Immunoblot to detect the K63-linked polyubiquitination of RORγt in Th17 cells. MG132 (2 μM) was added to prevent the proteolytic degradation of RORγt. RORγt was pulled down from MSC-sEVs-treated Th17 cell lysates using an anti-RORγt antibody. Immunoprecipitates were separated by SDS‒PAGE, transferred to PVDF membranes, and probed with anti-RORγt and anti-K63 ubiquitin antibodies. MSC-sEVs treatment destabilized RORγt through K63-deubiquitination.

To delineate the mechanisms by which MSC-sEVs reduced RORγt expression, the phosphorylation status of STAT3, the upstream regulator of RORγt, was first investigated by western blot analysis. As shown in Fig. 3A, MSC-sEVs treatment did not significantly change the phosphorylation status of STAT3 20 min after the initiation of Th17 differentiation, suggesting that MSC-sEVs regulate the expression of RORγt downstream of STAT3 phosphorylation. To determine whether MSC-sEVs regulate the expression of *Rorc* at the mRNA or protein level, MSC-sEVs were added to Th17 cells at various time points during the 5 days of Th17 differentiation, and the expression of *Rorc* mRNA and RORγt protein was measured. The normal expression kinetics of the RORγt protein in CD4^+^ T cells activated in the presence of Th17 polarizing cytokines over time (Supplementary Fig. 4) revealed that RORγt was detected at 24 h, peaked at 48 h, decreased at 72 h, and was maintained until 120 h. Interestingly, when MSC-sEVs were added at 5, 24, 48, 72, and 96 h following the onset of Th17 differentiation, the mean fluorescence intensity (MFI) of RORγt protein measured by flow cytometry analysis at 120 h was significantly decreased (Fig. 3B). The normal expression kinetics of *Rorc* mRNA measured by qRT‒PCR (Supplementary Fig. 4) revealed that the level of mRNA increased approximately twofold at 5 h following T-cell activation, peaked at 72 h, and reached a plateau after 120 h. Unlike the kinetics of the RORγt protein, the addition of MSC-sEVs did not alter the normal expression kinetics of *Rorc* mRNA (Fig. 3C), confirming that MSC-sEVs regulated the expression of RORγt at the protein level. To confirm and visualize the protein degradation of RORγt, cycloheximide (CHX) chase followed by western blot analysis was performed. MSC-sEVs and CHX were added 12, 24, and 48 h before Th17 differentiation was complete. Then, T cells were harvested at 120 h and subjected to western blotting. As shown in Fig. 3D, MSC-sEVs significantly reduced RORγt in the absence of de novo translated RORγt. A similar result was confirmed by flow cytometry (Supplementary Fig. 5). Intracellular cytokine staining of the MSC-sEVs-treated Th17 cells over time confirmed that the reduction in RORγt resulted in a decrease in IL-17 production (Fig. 3E). The ubiquitin‒proteasome system (UPS) is responsible for the breakdown of cellular proteins^23^. To confirm the hypothesis that the UPS mediates the reduction in RORγt by MSC-sEVs, MG132 was added as a proteasome inhibitor. As shown in Fig. 3F, MG132 restored the MSC-sEVs-mediated reduction in RORγt expression. As K63 polyubiquitination is known to regulate the stability of RORγt^27^, K63-linked polyubiquitination of RORγt was assessed in Th17 cells in the presence of MSC-sEVs using immunoprecipitation. MG132 (2 μΜ) was administered to prevent the degradation of RORγt. Indeed, K63-linked polyubiquitination of RORγt was dramatically reduced upon MSC-sEVs treatment (Fig. 3G), indicating that MSC-sEVs destabilized RORγt by K63 deubiquitination.

### Suppression of ubiquitin ligase and acetylase activity of p300 by MSC-sEVs-derived Eid3 resulted in the proteasomal degradation of RORγt

**Fig. 4:**
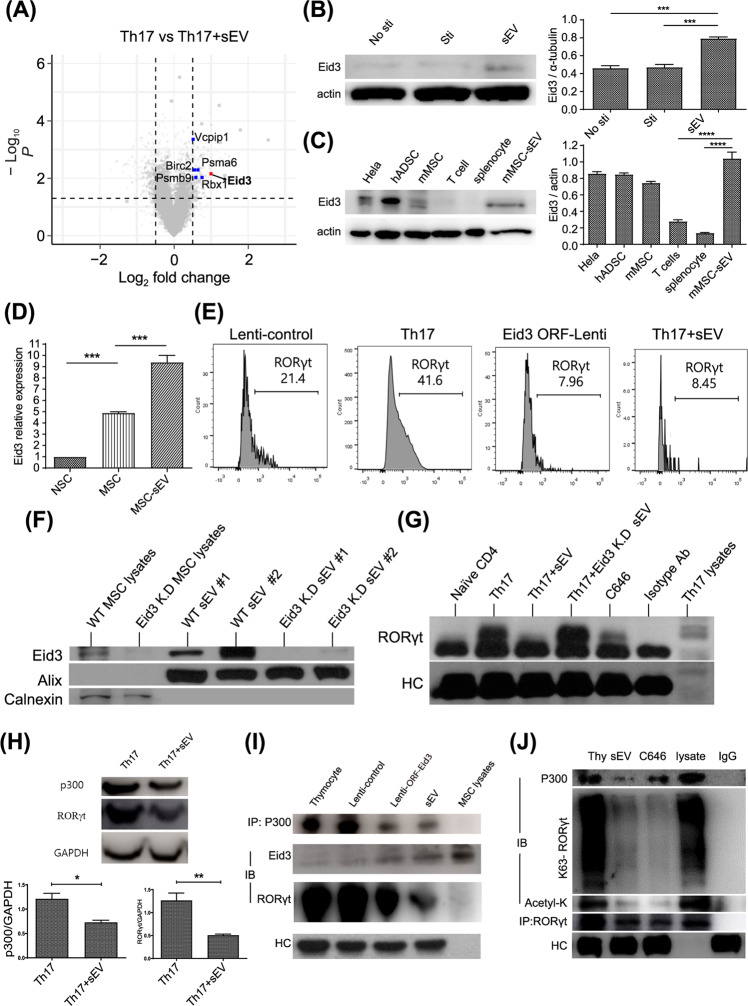
Eid3 from MSC-sEVs destabilized RORγt in Th17 cells. **A** Volcano plot of the differentially expressed mRNAs in MSC-sEVs-treated Th17 cells compared with untreated control Th17 cells. The dashed horizontal line indicates a *p* value of 0.05. The Eid3 gene, proteasome-related genes, and the other genes are colored red, blue, and gray, respectively. **B** The expression level of Eid3 in MSC-sEVs-treated T cells. The protein level of Eid3 was increased in MSC-sEVs-treated T cells. A representative image from three independent immunoblots is shown. The eid3/actin ratio is shown as a bar graph. No sti: without CD3/CD28 stimulation; Sti: with CD3/CD28 stimulation. ***p ≤ 0.001. **C** The protein level of Eid3 is expressed in each cell lysate. The relative expression of Eid3 in MSC-sEVs was the highest, and a negligible amount of Eid3 was observed in T cells and splenocytes, meaning that Eid3 was not intrinsic to activated T cells but came from MSC-sEVs. A representative image from three independent immunoblots is shown. The Eid3/actin ratio is shown as a bar graph. hADSC: human adipose-derived stem cell. ***p ≤ 0.001; ****p ≤ 0.0001. **D** mRNA level of Eid3 by real-time PCR (normalized by GAPDH). MSCs and MSC-sEVs both contained Eid3 mRNA. NSC: neural stem cell; MSC: mesenchymal stem cell. ***p ≤ 0.001. **E** Expression of RORγt after transduction of lentiviral Eid3-ORF (GFP-tagged) into Th17 cells (5 MOI). A lentiviral control-ORF particle (GFP-tagged) was used as a control (Lenti-control, 5 MOI). The downregulation of RORγt expression was also found in the lentiviral Eid3-ORF-transduced group. **F** The expression level of Eid3 in MSCs, Eid3-knockdown MSCs, MSC-derived sEVs, and Eid3-knockdown MSC-derived sEVs. Knockdown of Eid3 was performed by transducing the lentiviral Eid3-shRNA construct and was confirmed by western blotting. **G** Expression of RORγt in naive CD4+T cells, Th17 cells, MSC-sEVs-treated Th17 cells, Eid3-knockdown MSC-sEVs-treated Th17 cells, and C646-treated Th17 cells. The expression level of RORγt was assessed by immunoprecipitation with an anti-RORγt antibody. Eid3-knockdown MSC-derived sEVs failed to reduce the expression of RORγt. **H** Expression of p300 and RORγt in Th17 cells and MSC-sEVs-treated Th17 cells detected by western blotting. The expression levels of both p300 and RORγt were significantly decreased in MSC-sEVs-treated Th17 cells. A representative image from three independent immunoblots is shown. The p300/GAPDH and RORγt/GAPDH ratios are shown as bar graphs. *p ≤ 0.05; **p ≤ 0.01. **I** Regulation of p300 and RORγt by Eid3. Immunoprecipitation was conducted using an anti-p300 antibody, and immunoblotting was conducted using anti-p300, Eid3, and RORγt antibodies. The upregulated expression of Eid3 by lentiviral transduction or delivery of Eid3 through MSC-sEVs downregulated both p300 and RORγt. **J** Assessment of the polyubiquitination of K63 and the acetylation of RORγt by coimmunoprecipitation. Thymocytes treated with MSC-sEVs or C646 were immunoprecipitated using an anti-RORγt antibody. Immunoblotting was conducted using anti-p300, K63 polyubiquitin, acetyl-K, and RORγt antibodies. Lysate refers to thymocyte lysate without immunoprecipitation used as a positive control. Eid3 derived from MSC-sEVs destabilized RORγt by suppressing K63 polyubiquitination and acetylation through the inhibition of p300.

To identify the candidate regulators that degrade RORγt, proteasomal degradation-related genes differentially expressed between Th17 cells and MSC-sEVs-treated Th17 cells were analyzed by microarray analysis. Differentially expressed genes were categorized based on the Gene Ontology annotations using AmiGO 2 software. As indicated by the volcano plot in Fig. 4A, Eid3 (Ep300 interacting inhibitor of differentiation) was one of the highly expressed gene. Interestingly, Mamoru et al. reported Eid3 to be an indirect factor regulating proteasome activity^28^. The microarray data were validated by western blotting to determine whether the levels of Eid3 increased in the MSC-sEVs-treated T cells. As shown in Fig. 4B, the expression level of Eid3 was significantly increased in the MSC-sEVs-treated T cells. To establish whether Eid3 is intrinsic to activated T cells or derived from MSC-sEVs, Eid3 expression was assessed in cells from various lineages, including HeLa cells, human adipose-derived stem cells (ADSCs), murine MSCs, murine T cells, splenocytes, and murine MSC-sEVs, by western blotting. The relative expression of Eid3 was found to be highest in MSC-sEVs, as shown in Fig. 4C, and a negligible amount of Eid3 was observed in T cells and splenocytes, suggesting that Eid3 might be delivered to T cells via MSC-sEVs. Interestingly, MSC-sEVs also contained *Eid3* mRNA, as determined by real-time PCR (Fig. 4D, Supplementary Fig. 6). To confirm the contribution of Eid3 to RORγt degradation, naive CD4^+^ T cells were polarized to Th17 cells, and Eid3 expression was induced by transduction of a lentiviral construct encoding the Eid3-ORF. As shown in Fig. 4E, the forced expression of Eid3 downregulated RORγt, similar to MSC-sEVs. To determine whether Eid3 is sufficient for RORγt degradation, the expression of Eid3 was knocked down by transduction with the lentiviral Eid3-shRNA construct. Transduction of Eid3-shRNA into MSCs downregulated the expression of Eid3 in both MSCs and MSC-sEVs, as confirmed by western blotting (Fig. 4F). The MSC-sEVs isolated from the Eid3-shRNA-transduced MSCs failed to reduce the expression of RORγt, as shown in Fig. 4G. These results confirmed that Eid3 is necessary and sufficient for the downregulation of RORγt in Th17-polarized cells. Considering that Eid3 is a regulator of CBP/p300^29^, changes in the expression of p300 and RORγt were assessed in Th17-polarized cells in the presence of MSC-sEVs to investigate the association of p300 with RORγt. As shown in Fig. 4H, MSC-sEVs significantly decreased the expression of both p300 and RORγt.

To further identify the correlation of Eid3 with p300 and RORγt, coimmunoprecipitation was performed on thymocytes, which are known to express high levels of p300 and RORγt but not Eid3. Freshly isolated murine thymocytes were transduced with Eid3 ORFs containing lentiviral constructs or were treated with MSC-sEVs. The cell lysates were pulled down with anti-p300 antibodies and probed with anti-p300 Ab, anti-Eid3 Ab, and anti-RORγt Ab. Eid3 expression by lentiviral transduction or delivery of Eid3 through MSC-sEVs was found to downregulate p300 and RORγt, verifying the regulation of p300 and RORγt by Eid3 (Fig. 4I). Since p300 is known to stabilize RORγt through acetyltransferase and ubiquitin ligase activities, the polyubiquitination of K63 and the acetylation of RORγt were assessed by coimmunoprecipitation (Fig. 4J). Similar to C646, a p300 inhibitor, MSC-sEVs therapy significantly reduced both the polyubiquitination of K63 and acetylation of RORγt. Taken together, our results demonstrated that Eid3 derived from MSC-sEVs destabilizes RORγt by suppressing K63 polyubiquitination and acetylation through inhibition of p300.

### MSC-sEVs ameliorated symptoms of EAE in a murine model

**Fig. 5:**
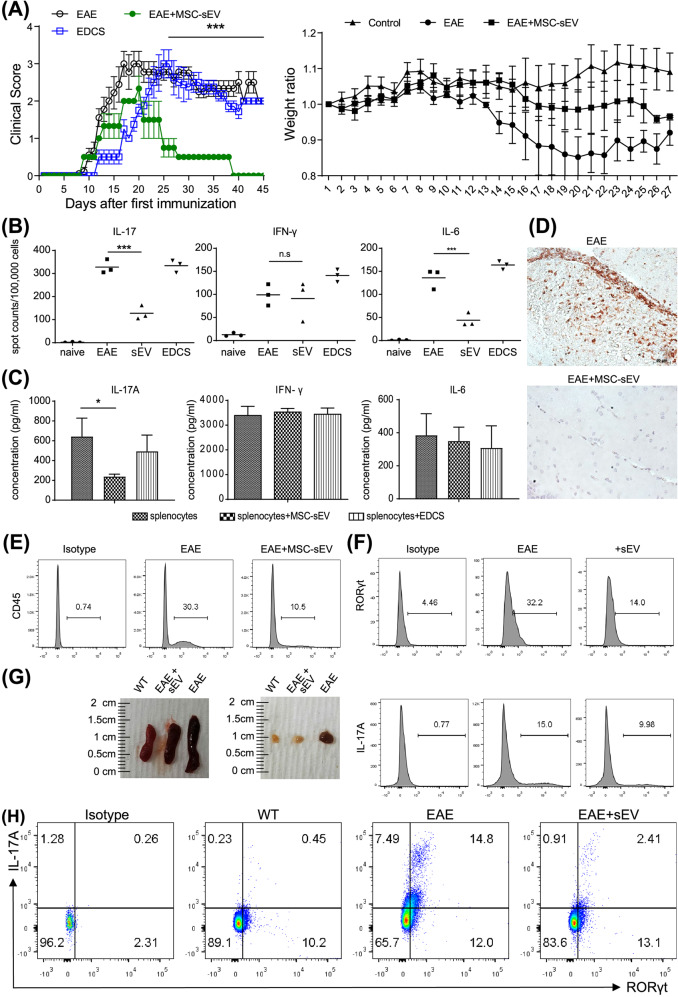
Injection of MSC-sEVs in mice ameliorates EAE. **A** Effect of MSC-sEVs treatment in a mouse EAE model. EAE was induced by MOG/CFA coinjected with pertussis toxin (PTx). Here, 25 μg of MSC-sEVs or sEVs-depleted supernatant (EDCS) was intravenously injected into EAE mice once a day for 7 days. Clinical scores of the diseased mice showed no change in the EDCS-treated group (*n* = 8) and a decrease in the sEVs-treated group (*n* = 6). The average body weights of the control, EAE mice, and EAE mice treated with MSC-sEVs were measured. The body weight increased in the control group (*n* = 3) and decreased in the EAE group (*n* = 3). Treatment with sEVs partially compensated for the weight loss from EAE (*n* = 3). ****p ≤ 0.0001. **B** Changes in the number of IL-17-, IFN-γ-, and IL-6-producing splenocytes (1 × 10^5^) from EAE mice analyzed by the enzyme-linked immunospot (ELISpot) assay. IL-17-producing splenocytes were significantly reduced in the MSC-sEVs-treated group but not in the EDCS-treated group. The means of triplicate experiments are shown, and the error bars indicate SEM. ***p ≤ 0.001. **C** Expression of secreted IL-17A, IFN-γ, and IL-6 from EAE-induced splenocytes (5 × 10^5^) restimulated with MOG measured by the Cytometry Bead Assay (CBA). IL-17 secretion by splenocytes was significantly reduced in the MSC-sEVs-treated group but not in the EDCS-treated group. *p ≤ 0.05. **D** H&E staining of mouse brain tissue filtration. Infiltration of CD3+ T cells was decreased in MSC-sEVs-treated EAE mice compared to EAE mice without MSC-sEVs treatment. **E** Flow cytometry analysis data showing a significant decrease in CNS tissue infiltrating CD45+ leukocytes in the MSC-sEV-treated group. **F** Flow cytometry analysis of the expression of RORγt and IL-17A in CNS-infiltrated CD4+ T cells. The expression of RORγt and IL-17A in CNS-infiltrated CD4+ T cells was downregulated in the MSC-sEVs-treated EAE group. **G** Size of the secondary lymphoid organs (spleen and lymph node) from WT, EAE, and EAE treated with the MSC-sEVs mice. **H** Analysis of the expression of RORγt and IL-17A among the CD4+ T cells in the draining lymph nodes from the WT, EAE, and EAE treated with the MSC-sEVs groups by flow cytometry. Compared to the control group, RORγt and IL-17A-producing CD4+ T cells were significantly decreased in the MSC-sEVs-treated EAE group.

A murine model for EAE, which is known to be a Th17-mediated disease, was used to test the effect of MSC-sEVs in vivo^30^. EAE was induced by MOG/CFA coinjected with pertussis toxin (PTx). MSC-sEVs (25 µg) or sEVs-depleted culture supernatant (EDCS) was administered intravenously to EAE-induced mice for 7 days. The clinical scores were tracked for 45 days before the mice were euthanized. For further investigation, immunohistochemical staining of the spinal cord and brain, as well as ELISpot and FACS analyses of the immune cells isolated from the spleen, draining LNs, and CNS tissue, were conducted. In contrast to EDCS, treatment with MSC-sEVs significantly lowered the clinical scores while maintaining body weight, as demonstrated in Fig. 5A. ELISpot analysis showed that IL-17-producing splenocytes were significantly reduced in the MSC-sEVs-treated group but not in the EDCS-treated group (Fig. 5B). Moreover, in an independent cytometric bead assay (CBA), IL-17 secreted from splenocytes was significantly reduced in the MSC-sEVs-treated group but not in the EDCS-treated group (Fig. 5C). Both the ELISpot and CBA assays showed that MSC-sEVs treatment had no effect on IFN-γ, indicating that MSC-sEVs specifically reduced IL-17 (Fig. 5B, C). Immunohistochemical staining showed that CD3^+^ T cells infiltrated the CNS of EAE-induced mice but not that of the MSC-sEVs-injected group (Fig. 5D, Supplementary Fig. 7). For further evaluation, the brain and spinal cord of the EAE-induced mice were procured, and the infiltrating immune cells were isolated using a Percoll gradient mononuclear cell isolation protocol^25^. FACS analysis of the isolated cells showed a substantial decrease in CNS-infiltrated CD45^+^ leukocytes in the MSC-sEVs-treated group (Fig. 5E). Importantly, the expression of RORγt and IL-17A in CNS-infiltrated CD4^+^ T cells was suppressed in EAE mice injected with MSC-sEVs (Fig. 5F). In general, the secondary lymphoid organs are enlarged in the inflammatory condition^31^. As shown in Fig. 5G, the spleen and draining lymph nodes were enlarged in the EAE-induced mice compared to the wild-type mice. However, the size of these organs in the EAE-induced mice treated with MSC-sEVs remained similar to that of wild-type mice. FACS analysis of the lymphocytes in the draining lymph nodes showed that RORγt and IL-17A-producing CD4+ T cells were significantly reduced in the MSC-sEVs-treated group compared to the untreated control group (Fig. 5H). Overall, these data showed that MSC-sEVs reduced the Th17-mediated immune response, hence alleviating the symptoms of EAE.

## Discussion

The anti-inflammatory and immunosuppressive effects of MSCs are attributed to their effects on lymphocytes and other cells involved in innate and adaptive immunity^32^. Similar to their parental MSCs, MSC-derived sEVs exert immunosuppressive effects by inhibiting the activation of immune cells and promoting the production of anti-inflammatory molecules, hence reducing inflammatory responses^33–35^. Studies have assessed the immense potential of MSC-derived sEVs as clinical therapeutics in various autoimmune disease models, such as experimental autoimmune encephalomyelitis (EAE)^36^, GvHD^37–39^, and acute kidney injury (AKI)^40^. Despite numerous studies demonstrating the immunomodulatory effects of MSC-derived sEVs, the underlying molecular mechanisms of immunosuppression by MSC-derived sEVs remain unknown. Previous studies have demonstrated that MSCs suppress the proliferation and activation of mouse T cells by secreting substances in culture^12,13^. While studying the immunomodulatory candidates secreted by MSCs, sEVs derived from MSCs were discovered to have a definite immunosuppressive effect^14^. Although previous studies have demonstrated the immunosuppressive effects of MSC-sEVs, the underlying mechanisms that mediate their suppressive function and whether the immunomodulatory effect of MSC-sEVs is specific to a particular lineage of T helper cells are still unclear.

This study discovered the molecular mechanism by which murine MSC-derived sEVs suppressed Th17 cells. Murine bone marrow-derived MSCs were cultured, and sEVs were isolated using differential ultracentrifugation. The immunophenotype of MSCs was positive for CD106, H2-Db, and CD44 but negative for CD34, CD14, CD11c, CD45, and I-Ab for >5 passages in our previous study^22^, confirming that they do not express hematopoietic stem cell markers and have a compatible phenotype to that of BM-originated MSCs. We did not directly compare our BM-MSCs with other MSCs (adipose-originated MSCs) in this study; however, unlike adipose-originated MSCs, our BM-MSCs were positive for CD106, which has been shown to be associated with immunomodulatory activity^41,42^. Characterization of sEVs showed that the isolated sEVs were lipid-bilayer vesicles that expressed exosomal markers such as CD9 and Alix and did not express the endoplasmic reticulum (ER) membrane-bound protein calnexin (Fig. 1B, C). The sucrose density gradient centrifugation showed that the range of specific density of sEVs corresponded to 1.15–1.19 g/ml (Fig. 1D). Confocal microscopy imaging and FACS analysis of the internalization of isolated sEVs suggested that at a given ratio of MSC-sEVs to CD4+ T cells, MSC-sEVs can be internalized by approximately 12% of T cells (Fig. 1F).

To investigate whether MSC-sEVs suppress the specific lineage of T cells, naive CD4^+^ T cells were induced to differentiate into Th17 cells, Th1 cells, and Tregs in the presence of MSC-sEVs. As a result, Th17 differentiation was specifically suppressed, and RORγt, a master transcription factor for determining the Th17 lineage, was reduced after MSC-sEVs treatment (Fig. 2A). In addition, IL-17A expression was specifically suppressed by MSC-sEVs but not other cytokines (Fig. 2B). This phenomenon was not detected when the sEVs-depleted culture supernatant (EDCS) was introduced to the differentiated T cells, indicating that Th17 cells and their cytokine-suppressing effects were exerted by sEVs but not by other factors in the culture media (Fig. 2C). In Fig. 2D, E, we sought to identify the effect of MSC-sEVs on the differentiation of T cells into Th17 cells independent of their suppressive effect on proliferation, as proliferation occurs ahead of differentiation and nonproliferating cells cannot differentiate. As such, when the MSC-sEVs were added 0 h after T-cell activation, T cells did not proliferate or differentiate, as shown in the right lower plot of Fig. 2D, E. When the MSC-sEVs were added 5 h after T-cell activation, 98% of cells proliferated in the Th17 differentiation condition, as shown in the middle lower plot of Fig. 2D. In the Th1 differentiation condition, approximately 95% of the cells proliferated. Therefore, the differentiation of each T-cell subset determined by the expression of master transcription factors among proliferated cells showed a significant reduction in Th17 cells (59–0%). The percentage of T-bet (+) cells was reduced from 25 to 17%, but the difference was not statistically significant. Aligned with this result, the production of IFN-γ was also not significantly reduced, as shown in Figs. 2B, 5B, and C. However, we could not exclude the possibility that MSC-EVs might contain biological activity to control T-bet expression, which should be investigated further.

We tested and compared MSC-derived sEVs with FBS-derived sEVs isolated from fetal bovine serum to determine whether the suppressive action of sEVs on Th17 cells is unique to MSC-derived sEVs. We induced the differentiation of naive CD4+ mouse T cells into Th1 and Th17 cells and treated them with FBS-sEVs. As a result, we found that there was no significant decrease in the expression of T-bet and IFN-γ in Th1 cells or RORγt and IL-17A in Th17 cells (Supplementary Fig. 3). Additionally, we confirmed that there was no expression of Eid3 mRNA in mouse NSCs (Fig. 4D and Supplementary Fig. 6). This result further demonstrates that the destabilizing effect of Eid3 on RORγt in sEVs can only be exerted by MSCs and not by other types of stem cells or sEVs.

The western blot data of STAT3 and its phosphorylated form showed no difference between MSC-sEVs treated and untreated Th17 cells, suggesting that MSC-sEVs regulate RORγt expression downstream of STAT3 (Fig. 3A). Moreover, measurements of RORγt protein and mRNA expression levels indicated that MSC-sEVs regulate RORγt expression at the protein level (Fig. 3B–D). Interestingly, this effect was abrogated by a proteasome inhibitor (MG132), implying that MSC-sEVs may mediate RORγt degradation at the posttranslational level (Fig. 3F, G). We noted that the MFIs of RORγt in Fig. 3B are not consistent with those in other figure. (Fig. 2D). We hypothesize that the difference in MFI values might be due to two factors. One is the limitation of the FACS analysis, and the other is the batch-to-batch variation in the biological activity of sEVs. In FACS analysis, we inevitably used various FACS machines, such as FACS Canto, Fortessa, X-20, and LSR2, installed in the core facilities of our institute, and these machines have different sensitivities for the detection of the same fluorescence. We consider that the different MFI values might reflect this limitation. For example, the baseline MFI of the negative control (no stimulation and isotype control) in Fig. 2D is almost zero; however, in Fig. 3B, it is approximately 800. The overall MFI values in Fig. 3D are higher than those in Fig. 2D. In addition, the compensation for the high concentration of CFSE dye in Fig. 2D made the overall fluorescence intensity in Fig. 2D lower. These limitations are described in the guidelines from the Euro-Flow consortium, which state that many aspects of flow cytometry experiments, including the FACS instrument, antibody panel, compensation settings, and antibody titration, may induce differences in MFI values^43,44^. For the batch-to-batch variation, we tried our best to control the quality and quantity variations of sEVs, for example, by controlling the passage number of source MSCs, standardizing the sEVs isolation protocols, and determining the quantity prior to use by ELISA and BCA as described in the Materials and Methods. However, currently, we do not have a way to control the batch-to-batch variation in biological activities. Once we identified the underlying mechanisms of the biological activities, we could then control the batch-to-batch variation in biological activities. In Fig. 3D and Supplementary Fig. 5, CHX was additionally used to inhibit further protein synthesis in differentiated Th17 cells. In this situation, CHX not only suppressed the translation of RORγt but also suppressed the translation of Eid3 mRNA contained in MSC-sEVs. This would result in a decrease in the overall proteolytic capacity of MSC-EVs, which contributed to the higher MFIs in Supplementary Fig. 5 compared to the MFIs in Fig. 2D. Additionally, the discrepancy might lie in the limitation of the FACS analysis and the batch-to-batch variation in the biological activity of sEVs, as mentioned above.

Studies have shown that the stability of RORγt is sustained by acetylation and K63-linked polyubiquitination^27,45,46^. In an experiment to determine whether MSC-sEVs may impact the acetylation or ubiquitination of RORγt, we discovered that Eid3 significantly reduced K63-linked polyubiquitination (Fig. 3G). Microarray analysis was conducted to examine how MSC-sEVs can affect the stability of RORγt via posttranslational modification. Eid3 was found to be highly expressed in MSC-sEV-treated Th17 cells (Fig. 4A). Eid3 is known as an inhibitor of p300 and a negative regulator of cell differentiation through binding with CBP/p300 or histone deacetylase (HDAC)^29,47^. According to Wang et al., p300 interacts with RORγt and binds specifically to the *IL-17* gene promoter interacting region (CNS2), conferring specificity to RORγt and promoting IL-17 expression^48^. Furthermore, p300 is known to have acetyltransferase and E3 and E4 ubiquitin ligase activities^21,29,49^. Interestingly, p300 has been reported to be a positive regulator of RORγt by promoting acetylation^46,50^. Therefore, our group focused on the role of Eid3 with p300 in Th17 cell differentiation.

We hypothesized that p300 would positively regulate RORγt via posttranslational modification, whereas Eid3 generated from MSC-sEVs would block this regulation. Eid3 was detected in MSC-sEVs but not in T cells or splenocytes, according to western blot analysis (Fig. 4C). In Th17 cells treated with MSC-sEVs, the expression of both p300 and RORγt was reduced (Fig. 4H). In addition, the expression of RORγt was downregulated in T cells overexpressing Eid3 (Fig. 4I). C646, a p300 chemical inhibitor, appeared to suppress both p300 and RORgt (Fig. 4J). These data implied that Th17 cells could be suppressed by Eid3 by downregulating p300. This finding correlates with the data that p300 selective inhibitors suppress Th17 cells^51^, as reported by Hammitzsch et al. Additionally, it was found that the knockdown of Eid3 in MSC-sEVs restored the stability of RORγt. Moreover, K63 polyubiquitination and acetylation of RORγt were suppressed by MSC-sEVs. Jain, S. and Wei, J. reported that the stability of p300 was entirely sustained by autoacetylation^52^. Thus, the degradation of p300 following treatment with MSC-sEVs or C646 may have been due to the inhibition of p300 autoacetylation by Eid3 or C646.

To confirm the activity and specificity of MSC-sEVs in suppressing Th17 immune responses in vivo, a murine EAE model was used. MSC-sEVs not only improved the clinical symptoms of EAE but also suppressed IL-17 secretion, which has been related to the pathogenicity of EAE and Th17 differentiation (Fig. 5A, B). In addition, the application of MSC-sEVs reduced the infiltration of CD45+ leukocytes (Fig. 5E). Moreover, RORγt and IL-17A expression in the CNS-infiltrated CD4+ T cells, IL-17A levels in draining lymph nodes, and the size of the spleen and lymph nodes were also decreased (Fig. 5F–H). Pachter et al. reported that human bone marrow-derived MSCs suppressed the differentiation of Th17 cells but not Th1 cells in the EAE model^53^. Although MSCs and MSC-derived extracellular vesicles alleviate the severity of EAE by suppressing Th17 cytokines^36^, Bial et al. showed that extracellular vesicles derived from MSCs improved experimental autoimmune uveitis by decreasing both pathogenic Th1 and Th17 cells^54^. Furthermore, adipose tissue-derived MSC-sEVs promoted Foxp3 expression in naive CD4^+^ T cells. In particular, RORγt and Foxp3 expression was increased when miR-10a was loaded into ADSC-derived MSC-sEVs^55^. Thus, the effects of MSC-sEVs on various types of T helper cells vary based on the experimental setting, such as the origin and environmental conditions of the MSCs. Therefore, it is important to note that research must emphasize controlling experimental conditions to verify the clinical feasibility of MSC-sEVs.

**Fig. 6:**
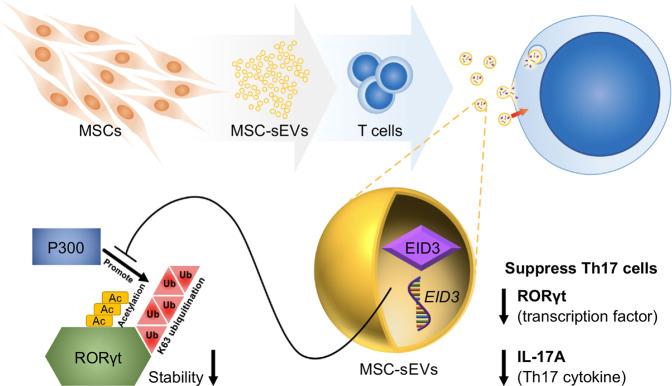
Schematic explanation of the underlying mechanism of MSC-derived sEVs in Th17 cell suppression. RORγt is stabilized by K63 polyubiquitination and acetylation by p300. Eid3 derived from MSC-sEVs suppresses the K63-linked polyubiquitination and acetylation of RORγt by p300, resulting in proteolytic degradation of RORγt. As a result, MSC-sEVs inhibit RORγt expression and IL-17 production in Th17 cells. MSC-sEVs exert their therapeutic effects by degrading RORγt at the protein level, resulting in the depolarization of Th17 cells. From this unique finding, MSC-sEVs are suggested to be useful in treating Th17-associated autoimmune diseases.

Many groups have reported the immunosuppressive effects of MSC-sEVs, suggesting that MSC-sEVs may be a potential candidate for cell-free therapy for a variety of autoimmune diseases. However, the factors that influence Th17 cells and their working mechanisms are not clear. Yang et al. reported that IFN-γ-primed MSC-sEVs attenuated colitis by suppressing Stat3, another Th17 transcription factor, specifically in Th17 cells via miR-125a,b^56^. miR-125a and miR-125b target *Stat3* mRNA, and their expression levels were significantly elevated after treatment with IFN-γ. Interestingly, MSCs that were not treated with IFN-γ did not express significant levels of miR-125a,b, but MSC-sEVs still promoted the specific suppression of RORγt and IL-17A. Furthermore, even following treatment with a miR-125a,b inhibitor, the expression of RORγt and IL-17A did not fully recover relative to the positive Th17 control group. This finding suggests the possible existence of RORγt targeting factors in MSC-sEVs. In this study, our group found that MSC-sEVs negatively regulated Th17 cells through Eid3. Interestingly, Xu et al. reported that Eid3 was highly expressed in MSCs and that a low level of Eid3 induced the transdifferentiation of MSCs into neural stem-like cells (uNSCLs)^57^. Thus, these findings strongly suggest that Eid3, which is highly expressed in MSCs, impacts cellular differentiation by regulating transcription factors and that Eid3 derived from MSC-sEVs could be used as a therapeutic agent to treat Th17 cell-associated autoimmune diseases. However, further studies are necessary to unravel the detailed underlying mechanisms by which the p300 region contributes to RORγt stability through K63 polyubiquitination and how Eid3 adversely controls p300 (Fig. 6).

Finally, we demonstrated that MSC-sEVs can exert their therapeutic effects by degrading RORγt at the protein level through K63-linked deubiquitination, resulting in the depolarization of Th17 cells in this study. This mode of action is similar to that of proteolysis-targeting chimeras (PROTACs), suggesting the potential of MSC-sEVs to be used as Th17 cell inhibitors. Ubiquitination-mediated proteolysis is the primary mechanism of protein homeostasis in cells. Recently, PROTACs, a promising therapeutic technology, have gained interest as a treatment for not only cancer diseases but also immunological disorders, viral infections, and neurodegenerative diseases^58^. In contrast to traditional small-molecule inhibitors that suppress the target protein by directly binding to the phosphorylation site, PROTACs regulate protein function by degrading proteins via E3 ligase, which mediates ubiquitin-mediated proteolysis. Because PROTACs function by binding to other sites and not the enzyme binding sites, they may provide a new approach to eliminate undruggable targets such as scaffold proteins, transcription factors, and protein aggregates that lack an active site for an enzyme. Based on our results of MSC-sEVs and the promising results of PROTACs, the mechanism of MSC-sEVs on Th17 cells could be applied to develop a new type of PROTAC for treating Th17 cell-related immune disorders. Taken together, the results of this study demonstrate that sEVs derived from MSCs could be a potential novel therapeutic agent for Th17-mediated autoimmune diseases.

## Supplementary information

Supplementary Figures

Z-axis serial sections of the confocal microscopic analysis revealed green fluorescence inside the T cells.

## Data Availability

All data generated or analyzed during this study are included in this published article and its supplementary information files.
