# Author Correction: Mesenchymal stem cell-derived extracellular vesicles subvert Th17 cells by destabilizing RORγt through posttranslational modification

**DOI:** 10.1038/s12276-023-01001-4

**Published:** 2023-04-26

**Authors:** Sunyoung Jung, Sunho Lee, Hyun Je Kim, Sueon Kim, Ji Hwan Moon, Hyunwoo Chung, Seong-Jun Kang, Chung-Gyu Park

**Affiliations:** 1 Department of Microbiology and Immunology, Seoul National University College of Medicine, Seoul, 03080 Korea; 2 Department of Biomedical Sciences, Seoul National University College of Medicine, Seoul, 03080 Korea; 3 Institute of Endemic Diseases, Seoul National University College of Medicine, Seoul, 03080 Korea; 4 Seoul National University Hospital, Seoul, Korea; 5 Transplantation Research Institute, Seoul National University College of Medicine, Seoul, 110-799 Korea; 6 Samsung Genome Institute, Samsung Medical Center, Seoul, Korea; 7 Cancer Research Institute, Seoul National University College of Medicine, Seoul, 03080 Korea; 8 BK21Plus Biomedical Science Project, Seoul National University College of Medicine, Seoul, 03080 Korea; 9 Biomedical Research Institute, Seoul National University College of Medicine, Seoul, 03080 Korea

**Keywords:** T cells, Mesenchymal stem cells

Correction to: *Experimental & Molecular Medicine* 10.1038/s12276-023-00949-7, published online 24 March 2023

After online publication of this article, the authors noticed an error in the author list, affiliation details, and acknowledgements section.

The correct statement of this article should have read as below.

In this article, the order that the authors appeared in the author list was incorrect.

The author list was incorrectly given as ‘Sunho Lee, Sunyoung Jung, Hyun Je Kim, Sueon Kim, Ji Hwan Moon, Hyunwoo Chung, Seong-Jun Kang & Chung-Gyu Park’ but should have been ‘Sunyoung Jung, Sunho Lee, Hyun Je Kim, Sueon Kim, Ji Hwan Moon, Hyunwoo Chung, Seong-Jun Kang & Chung-Gyu Park’.

In this article the affiliation details for Author Hyun Je Kim were incorrectly given as ‘Transplantation Research Institute, Medical Research Center, Seoul National University Hospital, Seoul, Korea’ but should have been ‘Transplantation Research Institute, Seoul National University College of Medicine, Seoul 110-799, Korea’.

In this article the Acknowledgements section was incorrectly given as ‘The authors thank Dr. Hyun-Je Kim and Yong-Hee Kim for their interpretation of the results’ but should have been ‘The authors thank Dr. Yong-Hee Kim for their interpretation of the results’.

The authors apologize for any inconvenience caused.

The original article has been corrected.

