# Supplementary material for: Mesenchymal stem cell-derived extracellular vesicles subvert Th17 cells by destabilizing RORγt through posttranslational modification
Source: Exp Mol Med. 2023 Mar 24;55(3):665–79. doi: 10.1038/s12276-023-00949-7 (PMC10073130; doi:10.1038/s12276-023-00949-7)
Supplement: Supplementary file 1 — Supplementary Figures [file 12276_2023_949_MOESM1_ESM.pdf]

Supplementary Material

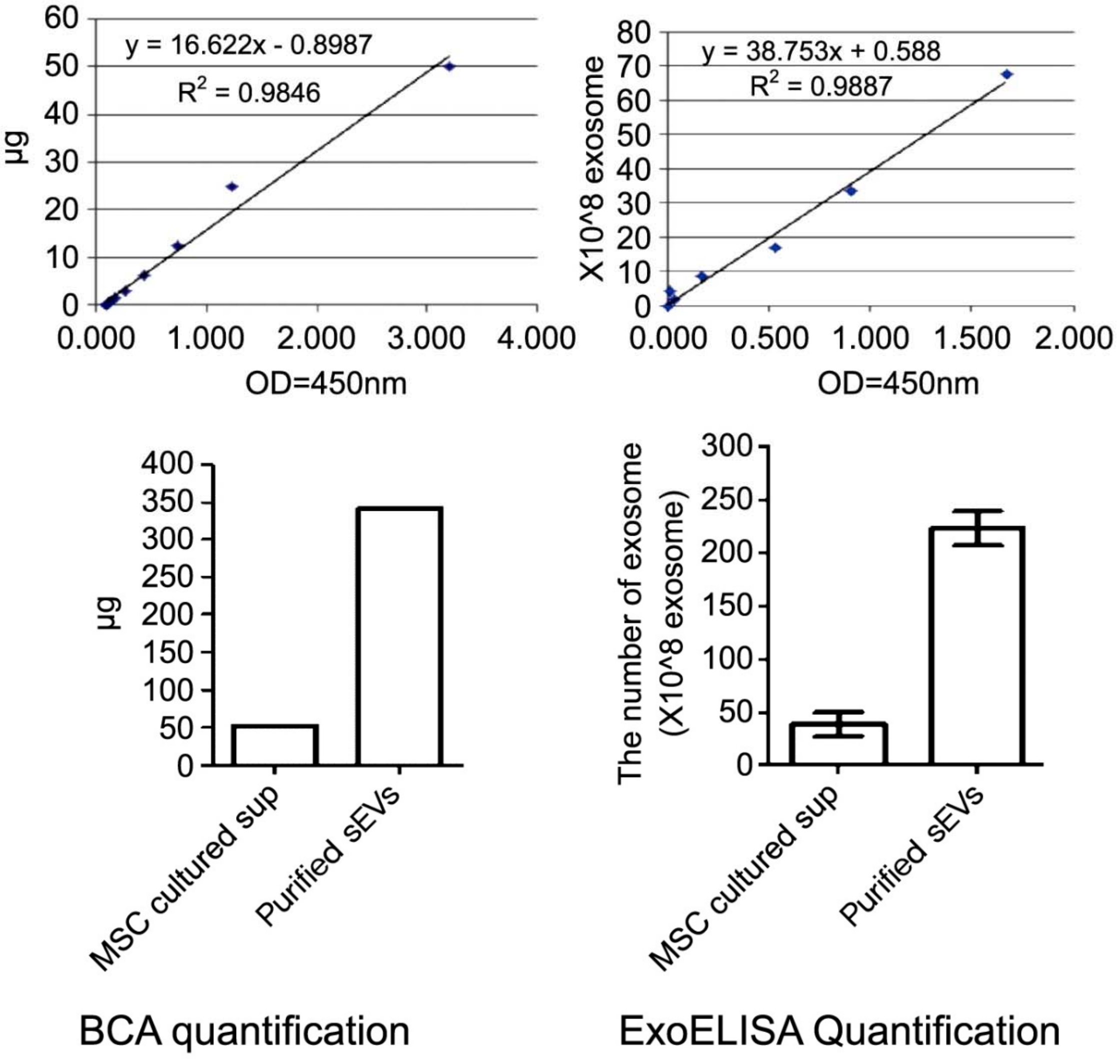

Supplementary Fig. 1. The total amount of proteins in the sEVs (341 μg) was quantified by BCA assay. The total number of sEVs (223×10^8) was calculated by ExoELISA.

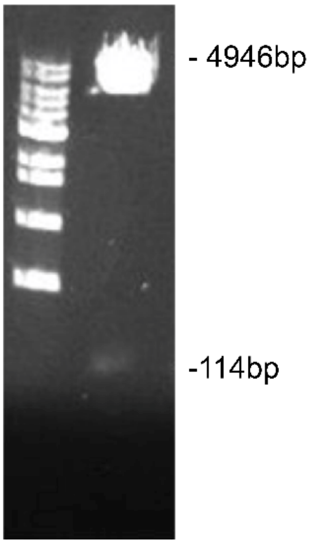

Supplementary Fig. 2. Lipid-raft GPI-anchored eGFP DNA bands were confirmed by gel electrophoresis. The lipid-raft GPI-anchored eGFP plasmid was restricted by restriction enzymes (Xba I, Apa I). The total plasmid band size (5060 bp) was divided into 4946 bp (eGFP region) and 114 bp (GPI region).

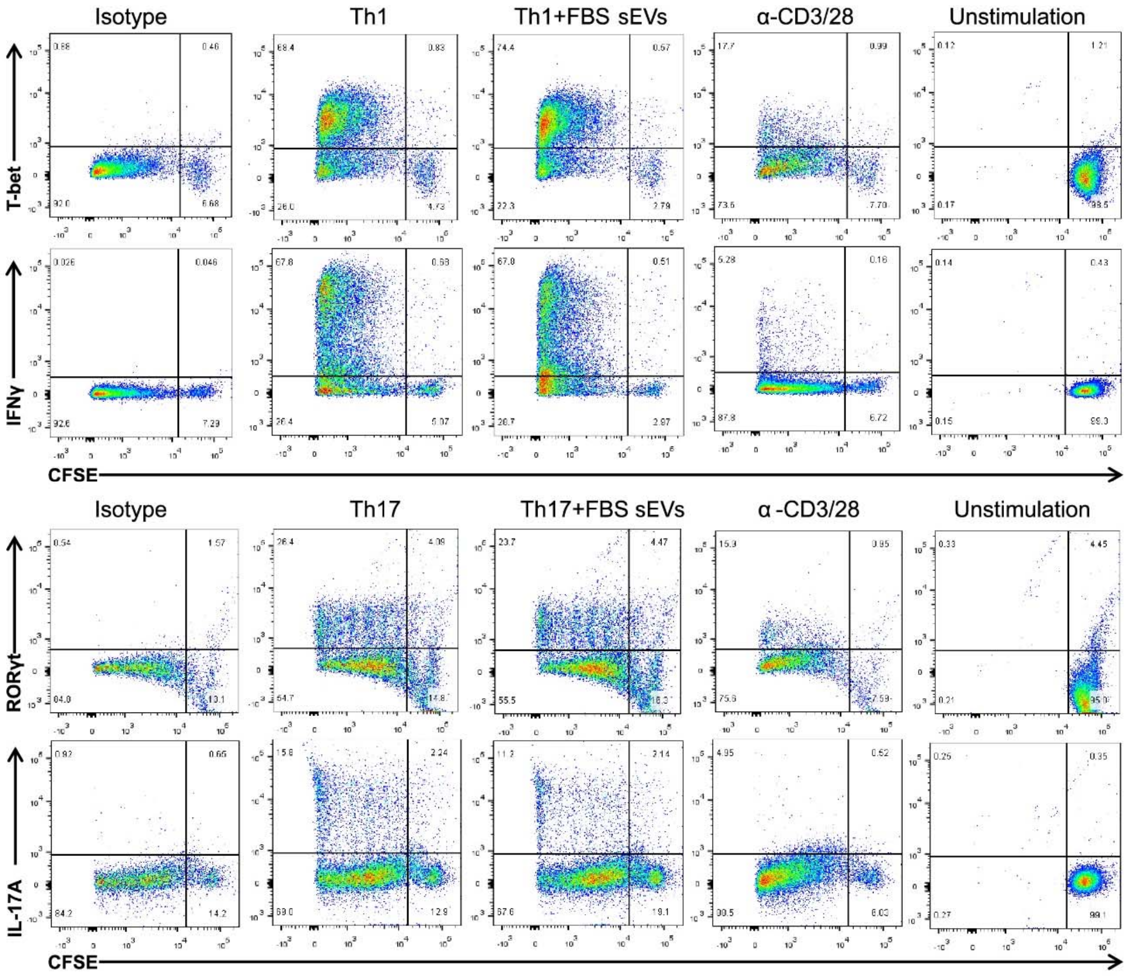

Supplementary Fig. 3. Naïve CD4 T cells were differentiated into Th1& Th17 cells and treated with fetal bovine serum (FBS)-control sEVs.

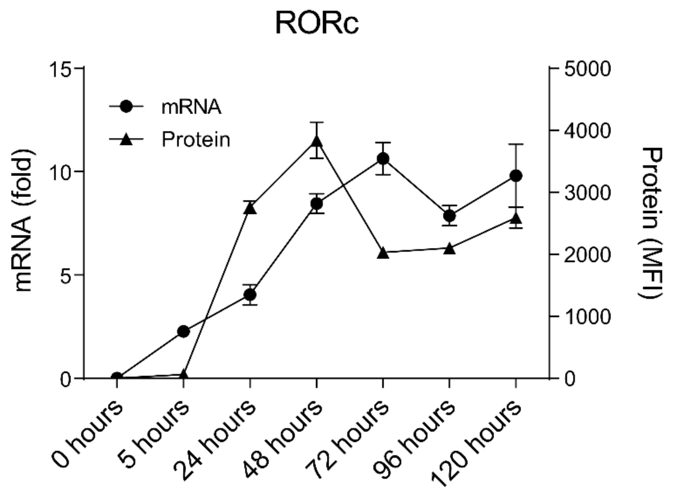

Supplementary Fig. 4. RORγt mRNA and protein expression in Th17 cells during in vitro differentiation performed for 5 days.

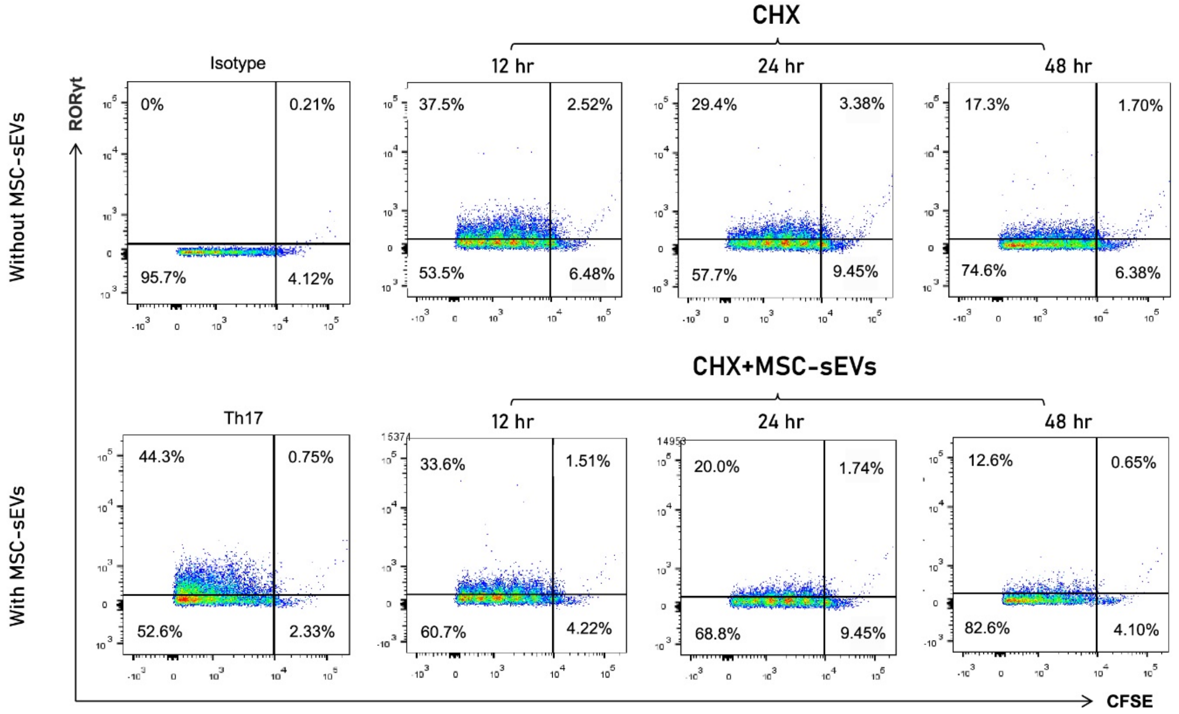

Supplementary Fig. 5. In each Th17 differentiation condition, 0.5 μg/ml cycloheximide was added at 12, 24, and 48 h before the end of the incubation (5 days) with sEVs.

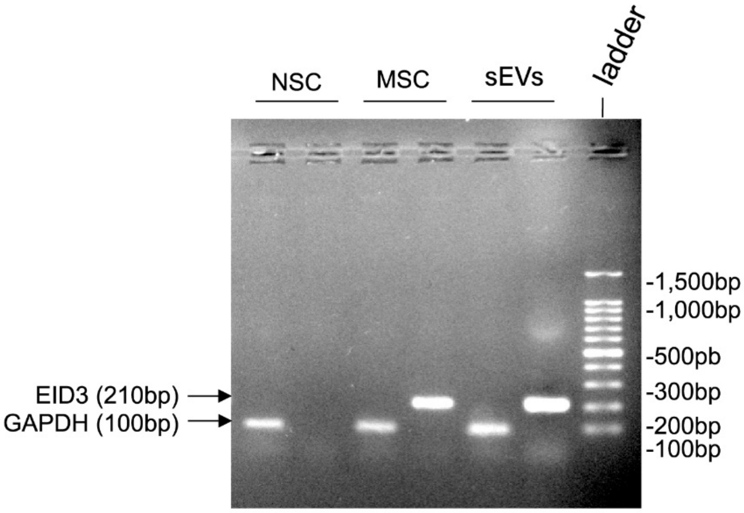

Supplementary Fig. 6. Expression of Eid3 was confirmed by a band on agarose gel after PCR (GAPDH housekeeping gene was used for normalization).

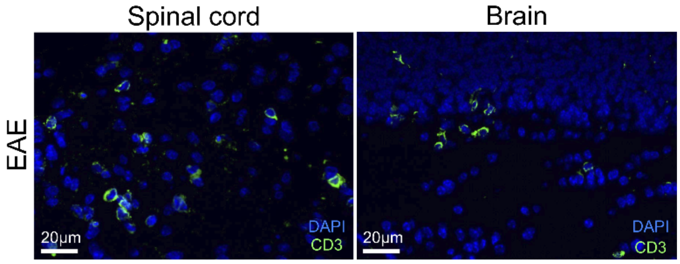

Supplementary Fig. 7. Immunohistochemistry of CNS-infiltrated CD3+ T cells (FITC = CD3, DAPI = Nucleus).

Supplementary Video 1. Z-axis serial sections of the confocal microscopic analysis revealed green fluorescence inside the T cells.
